# Nano‐omics: Frontier fields of fusion of nanotechnology

**DOI:** 10.1002/SMMD.20230039

**Published:** 2023-12-14

**Authors:** Xuan Wang, Weichen Xu, Jun Li, Chen Shi, Yuanyuan Guo, Jinjun Shan, Ruogu Qi

**Affiliations:** ^1^ School of Medicine & Holistic Integrative Medicine Nanjing University of Chinese Medicine Nanjing China; ^2^ Jiangsu Key Laboratory of Pediatric Respiratory Disease Institute of Pediatrics Nanjing University of Chinese Medicine Nanjing China; ^3^ Medical Metabolomics Center Nanjing University of Chinese Medicine Nanjing China; ^4^ Department of Nanomedicine Houston Methodist Research Institute Houston Texas US

**Keywords:** nano‐omics, nanotechnology, omics

## Abstract

Nanotechnology, an emerging force, has infiltrated diverse domains like biomedical, materials, and environmental sciences. Nano‐omics, an emerging fusion, combines nanotechnology with omics, boasting amplified sensitivity and resolution. This review introduces nanotechnology basics, surveys its recent strides in nano‐omics, deliberates present challenges, and envisions future growth.

## Graphical abstract

Nano‐omics, an emerging fusion, combines nanotechnology with omics, boasting amplified sensitivity and resolution. In this review, we introduce nanotechnology's basics, surveys its recent strides in nano‐omics, deliberates present challenges, and envisions future growth, and we summarize how nano‐omics as a fusion of nanotechnology and omics can be used to explore multiple pathways that could lead to transformative breakthroughs that enrich our ability to gain insight into the intricacies of life and health, including disease diagnosis, among others, although it may raise some safety and ethical issues.

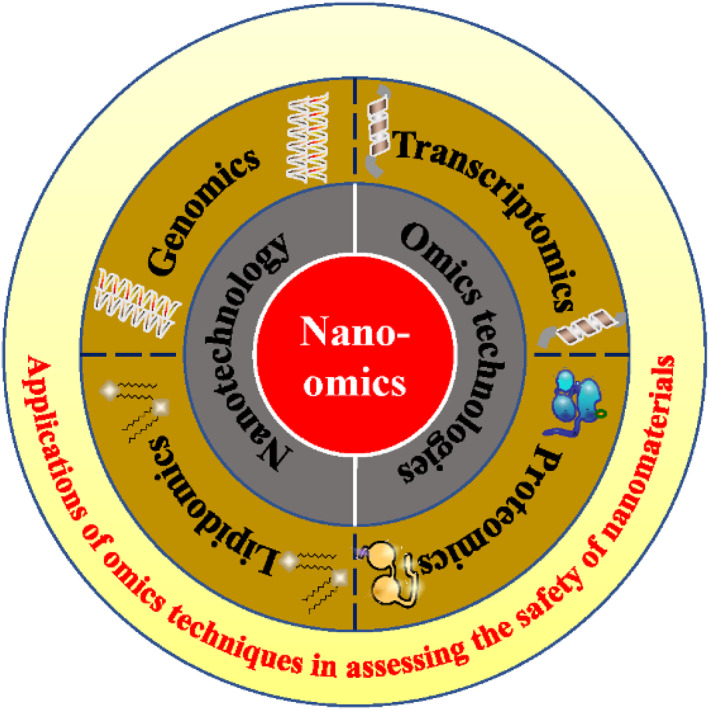

**Key points**

- Fundamental concepts of nano‐omics are introduced.
- Applications of the integration of nanotechnology and omics technologies are presented.
- Future potential and remaining challenges of nano‐omics are discussed.

## 1 INTRODUCTION

Omics includes a variety of sub‐disciplines, including genomics, transcriptomics, metabolomics, and proteomics, among others, to provide a complete picture of the molecular landscape.^1^, ^2^ The goal of omics is to acquire a comprehensive comprehension of intricate biological mechanisms at the molecular scale, thereby offering insights beyond the scope of conventional methodologies. This multidisciplinary approach explores the composition, structure, function, and interactions of specific molecules, creating a more holistic perspective of biological systems. Thus, omics relies on collective high‐throughput technologies that allow researchers to gather and analyze large amounts of molecular data efficiently.

**FIGURE 1:**
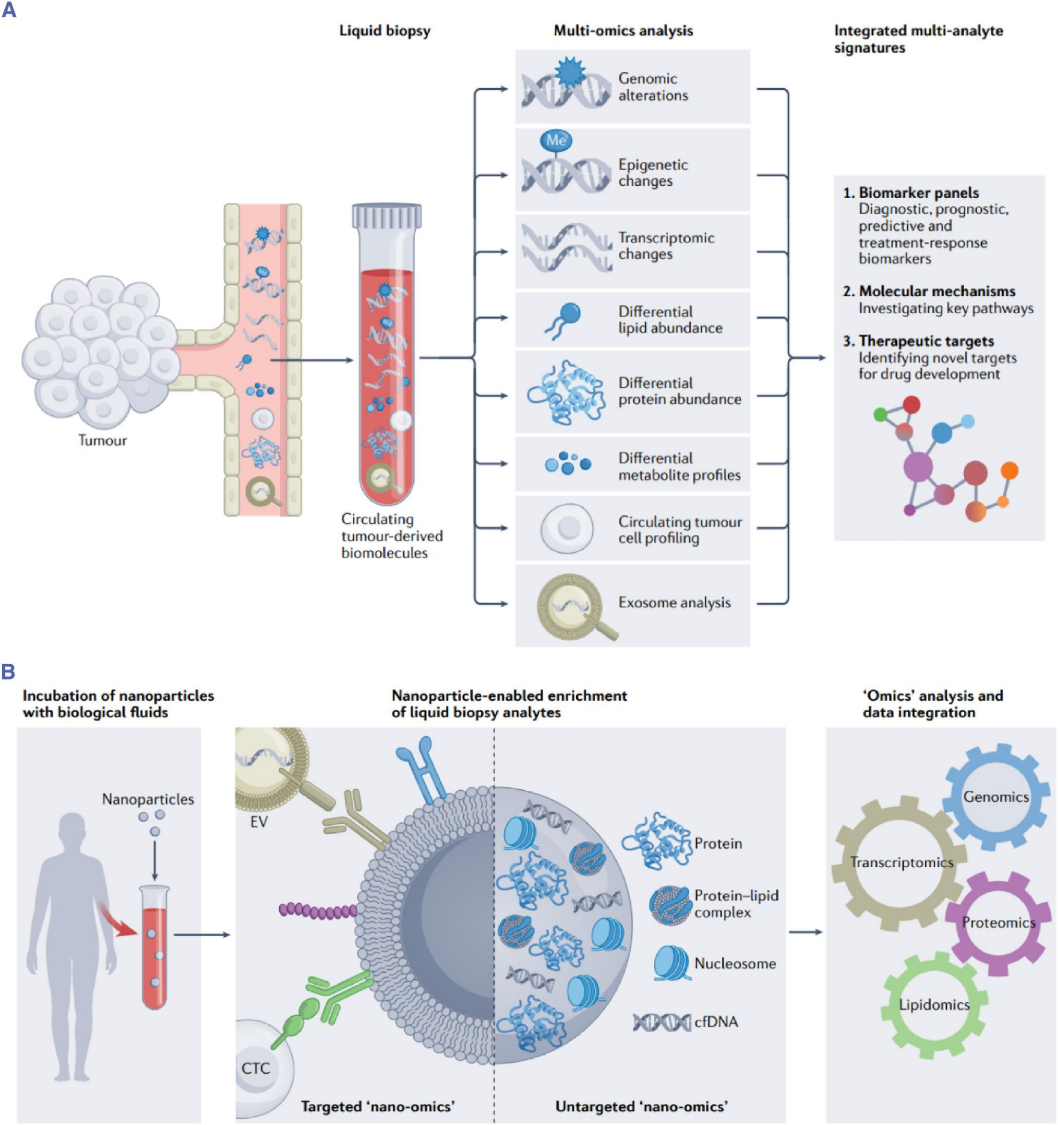
(A) The nano‐omics paradigm. Translational potential of multi‐omics liquid biopsy. (B) Nanomaterial‐based harvesting platforms can simultaneously enrich cancer‐specific genomic, transcriptomic, proteomic and lipidomic signatures from a single peripheral blood sample (and potentially other biological fluids). The nano‐omics approach seeks to apply the knowledge garnered at the bio‐nano interface in order to enable the multi‐omics analysis of complex biological fluids, with the ultimate goal of unveiling novel multi‐analyte biomarker panels for early cancer detection. *Source*: Reproduced with permission.^3^ Copyright 2022, Springer Nature Limited.

Nanotechnology has revolutionized omics research by allowing scientists to explore even deeper into the microscopic world. This field utilizes nanoscale manipulations to extract finer details from biological systems. Nanotechnology's capabilities to manipulate and interact with biomolecules at the microscale have enabled heightened precision, ensuring that researchers can extract more information from biological systems. The integration of nanotechnology and omics has led to the creation of a new field of research, known as nanoomics, which involves using nanotechnology to isolate and analyze substances from biological fluids for subsequent omics research and analysis (Figure 1).^3^ Nanoomics has brought about transformative advancements in the study of diseases. The nuanced insights gleaned from nanoomics analyses pave the way for tailored therapeutic approaches, where treatments are customized based on individual patient profiles. This transition from a one‐size‐fits‐all model to precision medicine marks a fundamental change in healthcare, promising more effective and less invasive interventions.

In conclusion, the fusion of nanotechnology and omics epitomizes the synergy between scientific frontiers. This fusion propels us into an era of deeper understanding and more accurate manipulation of biological systems. As we navigate the intricate realm of molecules within living organisms, nanoomics holds the promise of revolutionizing fields as diverse as medicine, environmental science, and agriculture, paving the way for novel solutions and transformative discoveries.

## 2 THE DEVELOPMENT PROCESS OF NANOTECHNOLOGY

The inception of nanotechnology dates back to 1959 when Richard Feynman, an American theoretical physicist, introduced the concept during a meeting of the American Physical Society at the California Institute of Technology.^4^ As this technology continues to advance, the emergence of various advanced microscopes capable of observing particles at the nanoscale has been a critical development. Notably, these microscopes include Scanning Tunneling Microscopy (STM)^5^ and Atomic Force Microscopy (AFM),^6^ both of which are Scanning Probe Microscopes (SPMs).^7^ These instruments have enabled the meticulous observation of particle structures at the nanoscale, unlocking new possibilities in material preparation and manipulation. Using scanning probe microscopy, individual molecules adsorbed on the surface can be probed with ultra‐high resolution at low temperatures and under ultra‐high vacuum to determine the details of their structure and their contribution to conformation, configuration, charge states, aromaticity, and resonance structure.^7^ This achievement represents a momentous landmark in the realm of nanotechnology and its convergence with microscopy, heralding the advent of a fresh and transformative epoch of investigation on the nanoscale. The strides made in this direction underscore the remarkable synergy between these two disciplines, offering unprecedented opportunities for investigating the intricacies of matter at its smallest dimensions. The convergence of nanotechnology and microscopy has produced a paradigm shift that allows researchers to navigate the complex landscape of atoms and molecules. This symbiotic relationship enables the visualization and manipulation of materials with unparalleled precision, opening windows to realms previously inaccessible. The implications span across various sectors, from material science to medicine, and promise breakthroughs that were once deemed inconceivable.

**FIGURE 2:**
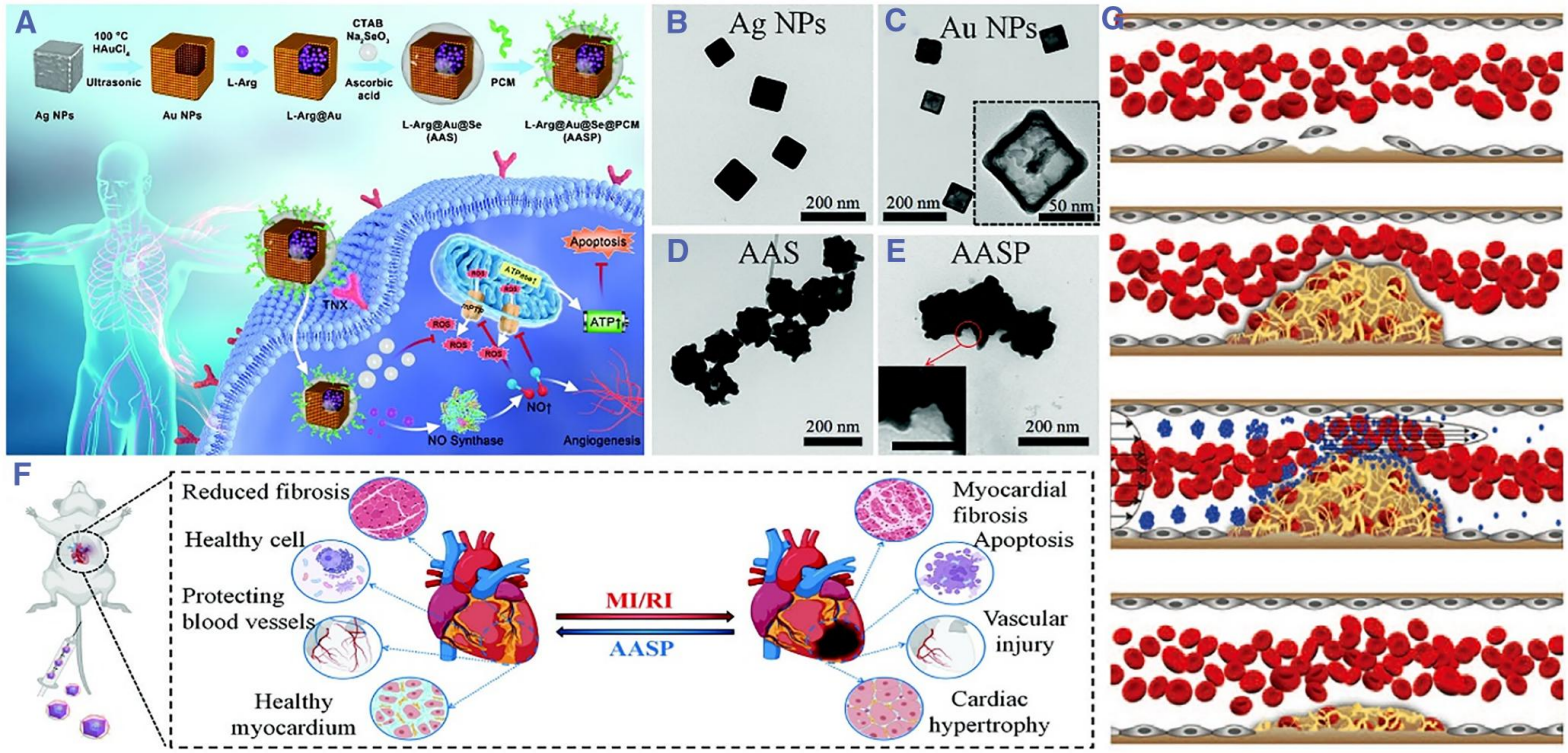
(A) Synthesis route and mechanism underlying the effects. L‐Arg‐loaded gold nanocages ameliorate myocardial ischemia/reperfusion injury by promoting nitric oxide production and maintaining mitochondrial function. TEM images of Ag nanoparticles (B), Au nanocages (C), AAS (D) and AASP (E). (F) Schematic representation of cardiac changes in rats before and after AASP treatment of MI/RI. *Source*: (A‐F) Reproduced under terms of the CC‐BY license.^9^ Copyright 2023, The Authors, published by Wiley‐VCH GmbH. (G) Schematic representation of the experimental strategy. Ferric chloride injury initiates the formation of a thrombus (top) that grows to partially obstruct blood flow (top middle). Intravenously injected SA‐NTs dissociate into NPs at the thrombus site because of the rise in local shear stress (bottom middle). Accumulation of tPA‐coated NPs and binding to the clot at the occlusion site progressively dissolve the obstruction (bottom). *Source*: Reproduced with permission.^10^ Copyright 2012, The American Association for the Advancement of Science.

Nanotechnology is a multidisciplinary field focused on understanding and utilizing material properties at the nanoscale, typically ranging from 1 to 100 nm^8^ Since its inception in the 1980s, nanotechnology has experienced gradual development, including diverse domains including materials science, biology and medicine. Among its numerous applications, nanotechnology's potential in the realm of biomedicine has been particularly notable. One of its prominent applications is the utilization of nanoparticles (NPs) as versatile carriers for targeted drug delivery and controlled release, enhancing the efficiency and precision of medical treatments (Figure 2).^9^, ^10^, ^11^

**FIGURE 3:**
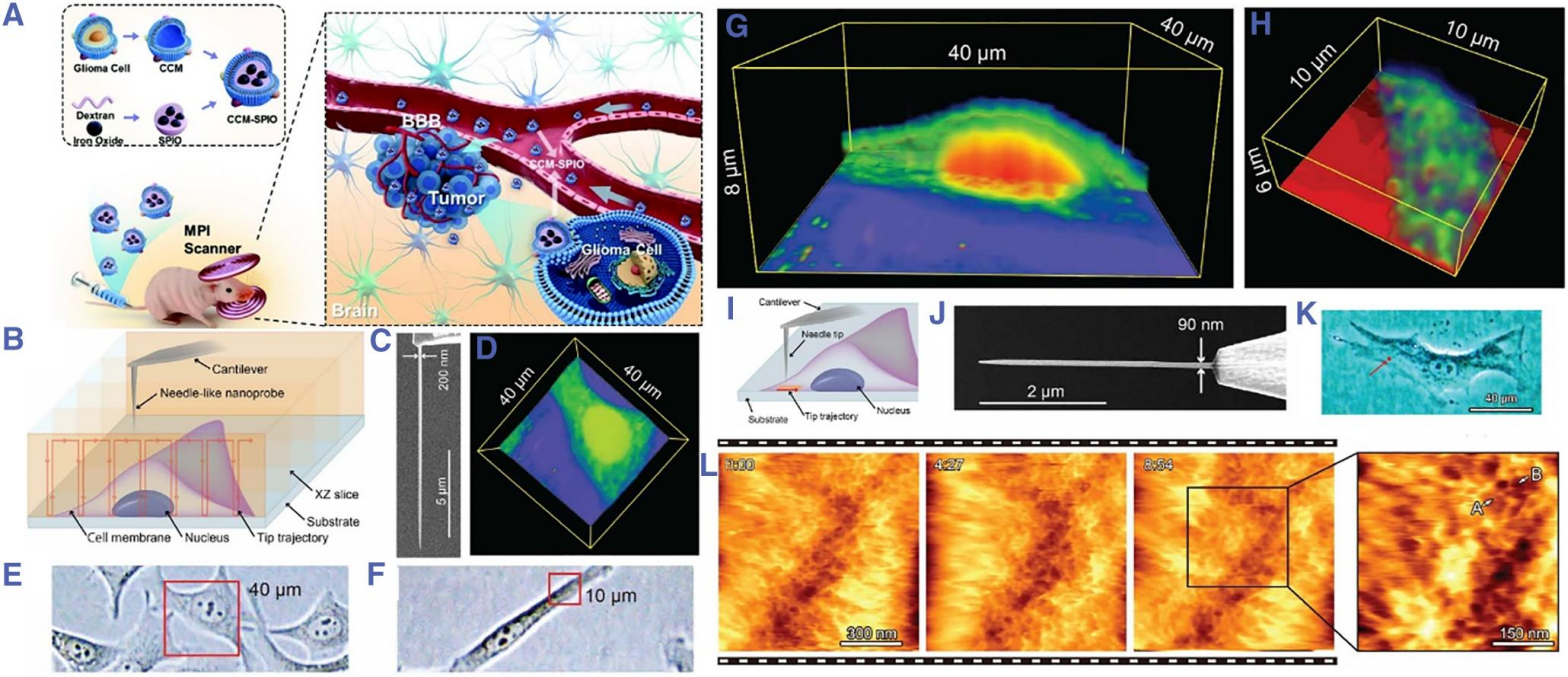
(A) Schematic illustration of the synthetic and functional process of CCM‐SPIO nanoprobes. *Source:* Reproduced under terms of the CC‐BY license.^15^ Copyright 2023, The Authors, published by Wiley‐VCH GmbH. (B) 3D nanoendoscopy‐AFM technique. Schematic illustration of the 3D nanoendoscopy‐AFM method, where the nanoprobe is repeatedly introduced inside the cell at different positions in the desired area. (C) FIB‐fabricated nanoprobe used in the 3D nanoendoscopy‐AFM measurements. (D) 3D nanoendoscopy‐AFM cell map of the entire HeLa cell volume (40 × 40 × 6 μm^3^) enclosed in the red square in (E), where the cell membrane, nucleus, and cytoplasmic regions can be distinguished in the cross section displayed in (G). (H) 3D nanoendoscopy‐AFM image of a HeLa cell volume (10 × 10 × 6 μm^3^) enclosed in the red square in (F), where internal granular structures can be clearly recognized. (I) 2D nanoendoscopy‐AFM technique. An illustration of the 2D nanoendoscopy‐AFM method, where the nanoprobe is inserted inside the cell to measure the cytoplasmic side of the cell membrane using amplitude modulation mode AFM. (J) Example of an EBD‐fabricated nanoprobe used in 2D nanoendoscopy‐AFM, where the length of the needle should be long enough to completely penetrate the cell and reach its bottom part and thin enough to reduce the cell damage. (L) Consecutive 2D nanoendoscopy‐AFM 1 μm × 1 μm images performed on a BALB/3T3 fibroblast on the region highlighted by the red dot depicted in (K), showing the reticular structure of the inner surface of the cell membrane forming its scaffolding and also the mem‐brane fluctuations during the measurements. Zoomed area of the images displayed in (L). *Source:* (B‐L) Reproduced with permission.^16^ Copyright 2021, The Authors, published by American Association for the Advancement of Science.

**FIGURE 4:**
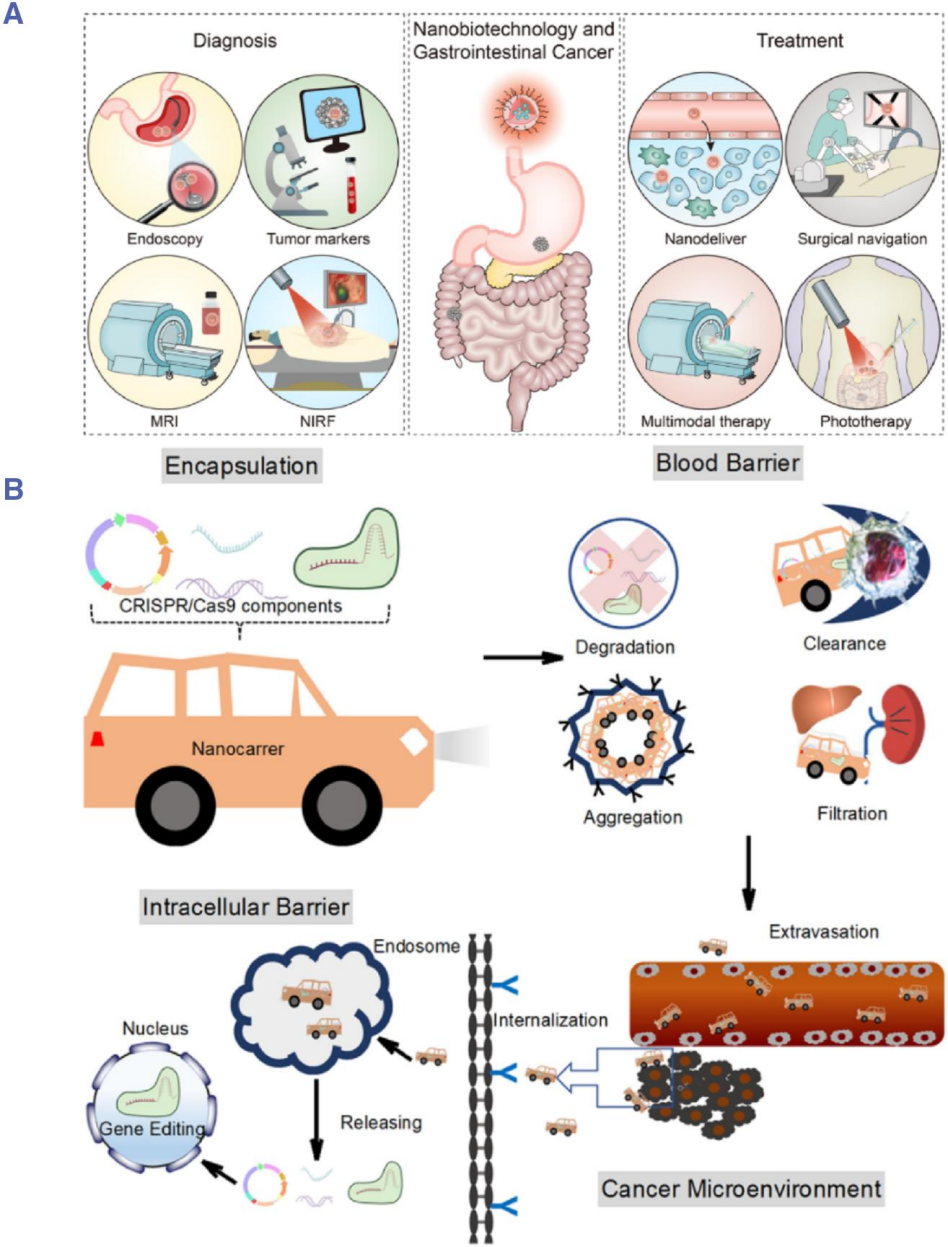
(A) Application of nanotechnology in the diagnosis and treatment of gastrointestinal cancer. *Source:* Reproduced under terms of the CC‐BY license.^18^ Copyright 2022, The Authors, published by BioMed Central. (B) Schematic illustration of different barriers in the process of CRISPR/Cas9 delivery for cancer therapy. *Source:* Reproduced under terms of the CC‐BY license.^19^ Copyright 2021, The Authors, published by Elsevier B.V.

Nanotechnology has also significantly influenced cellular imaging and localization.^12^, ^13^, ^14^ Nanoscale imaging techniques have paved the way for scrutinizing intricate cellular structures with unprecedented resolution, yielding insights into the dynamics of biological processes (Figure 3).^15^, ^16^, ^17^ Furthermore, nanotechnology has begun to revolutionize clinical medicine. In this context, nanotechnology has contributed to the early diagnosis and treatment of diseases, and cancer treatment stands out as a prime example. Nanoparticles have been ingeniously designed to target cancer cells specifically, enhancing drug delivery while minimizing damage to healthy cells, groundbreaking advancement in oncology (Figure 4).^18^, ^19^, ^20^ The integration of nanotechnology and medicine paves the way for the future of healthcare and personalized and precise treatments. In essence, the journey of nanotechnology since the 1980s has propelled it into a remarkable realm, holding immense transformative potential across diverse scientific and medical domains. Its ability to utilize materials at the nanoscale not only deepens our understanding of fundamental mechanisms, but also facilitates a paradigm shift in the medical field. This shift holds the promise of revolutionizing medical practices, ushering in a new era of more targeted and efficacious treatments for a wide spectrum of health conditions.

## 3 INTEGRATION OF NANOTECHNOLOGY AND OMICS TECHNOLOGIES

### 3.1 Nanoomics in genomics and transcriptomics

**FIGURE 5:**
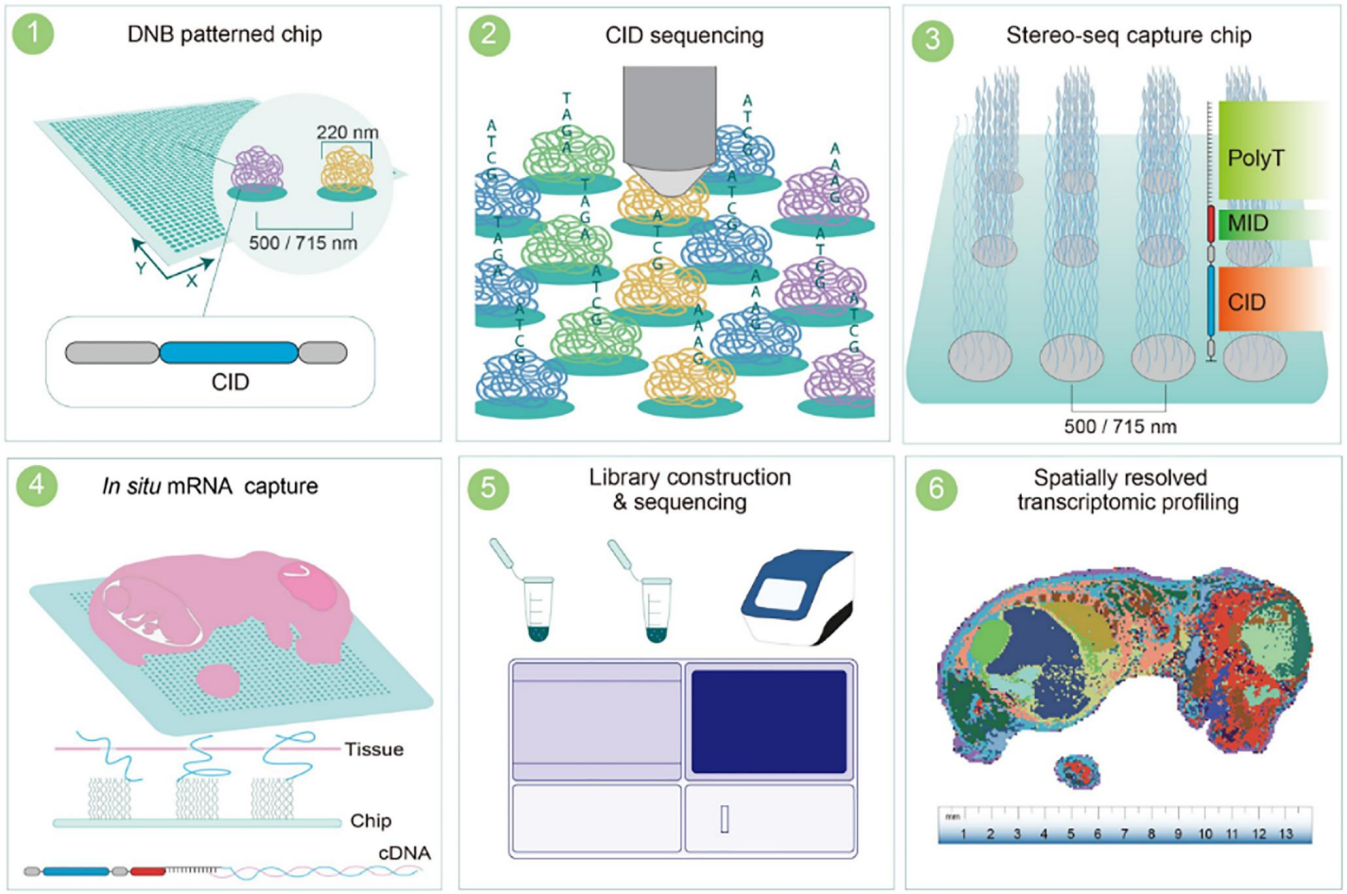
Schematic representation of the Stereo‐seq procedure. Step 1: Design of the DNB patterned array chip. Step 2: In situ sequencing to determine the spatial coordinates of uniquely barcoded oligonucleotides on the chip. Step 3: Preparation of Stereo‐seq capture probes by ligating the MID‐polyT containing oligonucleotides to each spot. Step 4: subsequent in situ RNA capture from tissue placed on the Stereo‐seq chip. Step 5: cDNA amplification, library construction and sequencing. Step 6: Data analysis to generate the spatially resolved transcriptome of the profiled tissue. *Source:* Reproduced under terms of the CC‐BY license.^22^ Copyright 2022, The Authors, published by Elsevier.

The synergistic fusion of DNA nanoball chips (DNBs) and in situ RNA capture technology has propelled the emergence of an innovative spatial transcriptomics approach characterized by nanoscale precision. This pioneering approach ushers in a new era of transcriptomic analysis, revealing the complex molecular landscape within cells with unprecedented resolution. This transformative technology, often hailed as nano‐transcriptomics, holds immense promise in transforming our understanding of cellular dynamics and functions. At the heart of this breakthrough lies the intricate marriage between DNBs and in situ RNA capture technology. DNBs, meticulously designed nanostructures, exhibit unique characteristics that enable them to capture and immobilize RNA molecules within cellular contexts. This strategic integration allows researchers to extract and preserve RNA signatures within their native cellular environment, avoiding the biases and artifacts that traditional extraction methods can generate.^21^ The significance of this approach cannot be overstated, as it not only enhances the accuracy of analysis but also offers the unprecedented advantage of visualizing the intricate spatial distribution of RNA molecules within cells. A distinguishing hallmark of nano‐transcriptomics is its ability to unveil subcellular‐level details. By capturing RNA molecules in situ, this technique reveals the transcriptomic nuances of distinct cellular compartments. This previously unattainable granular view enables the identification of cell‐to‐cell variants and subsets within tissues, enhancing our grasp of cellular heterogeneity. This subcellular resolution bridges the gap between genomics and cell biology, offering insights into the molecular underpinnings of cellular functions (Figure 5).^22^ Nanopore DNA sequencing and RNA sequencing has ignited a transformative revolution in genomic research, driven by their inherent advantages of ultra‐long sequencing read lengths and remarkable sequencing speed. These techniques break the limitations of tradition and provide a new paradigm for deciphering the complex genetic makeup of living organisms.^23^, ^24^, ^25^, ^26^, ^27^ Oxford Nanopore Technologies (ONT) long reads have been used to characterize complex genome rearrangements in individuals with genetic disorders. For example, ONT sequencing of the human genome showed that amplification of tandem repeats in the ABCA7 gene was associated with an increased risk of Alzheimer's disease.^28^

**FIGURE 6:**
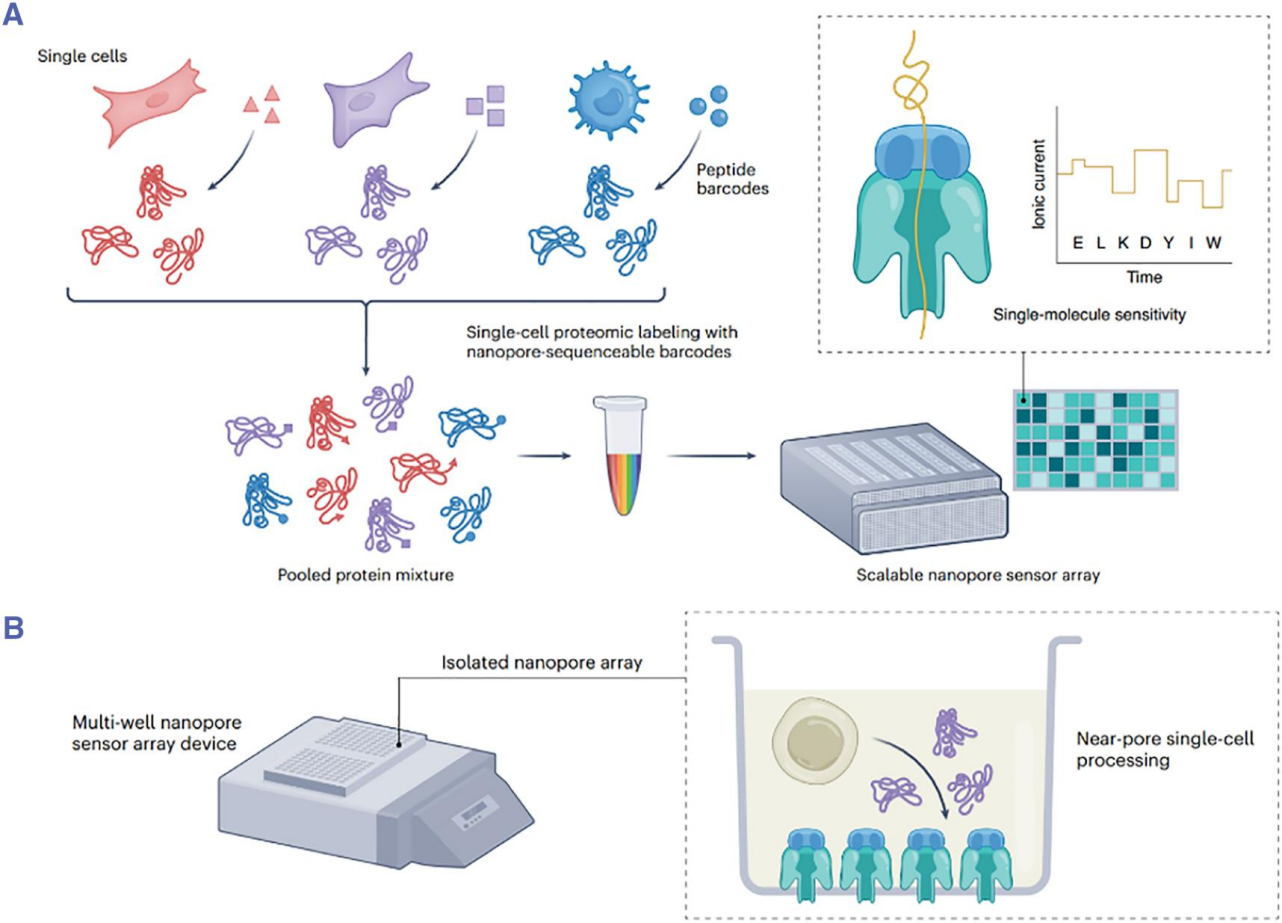
(A) Single‐cell nanopore proteomics with highly multiplexable peptide sequence‐based barcoding and highly parallel nanopore instruments. Multiplexity: protein samples isolated from single cells could be barcoded with peptide‐based barcodes for high‐throughput multiplexing. Sensitivity, throughput, scalability: motor‐mediated translocation of a single biopolymer such as DNA, RNA, or protein produces stepwise current signals that reflect the sequence of the biopolymer traversing the pore constriction. Nanopore sensors can be parallelized within a single flow cell and multiple flow cells across a single instrument. (B) Multi‐well plate‐based nanopore sensor arrays could enable well‐based single‐cell sample processing steps to occur nearer the nanopore sensors, potentially mitigating sample loss and increasing sensitivity. *Source:* Reproduced with permission.^30^ Copyright 2023, Springer Nature America.

Nanopore sequencing capitalizes on nanoscale pores, akin to minuscule channels, which allow individual DNA or RNA molecules to pass through.^29^ As these molecules pass through the nanopore, their unique molecular signatures induce distinct electrical currents, enabling real‐time sequencing of nucleotides. This real‐time analysis enables the acquisition of unprecedentedly long sequencing reads, far surpassing the capabilities of traditional sequencing methods. This extension in read length facilitates the assembly of complex genomes and enables the identification of structural variations that might have previously gone unnoticed. The rapidity of nanopore sequencing is equally astonishing. Traditional sequencing approaches involve laborious sample preparation and amplification steps, consuming significant time. In contrast, nanopore sequencing's streamlined methodology expedites the process, enabling the rapid generation of sequencing data. This swift turnaround is particularly advantageous for applications requiring timely results, such as clinical diagnostics or pathogen surveillance. The synergy between nanopore sequencing and genomics is indisputably transformative. Long‐read sequencing unravels the intricacies of genomes, unveiling previously obscured genomic regions and aiding in the understanding of genetic variations and hereditary diseases. In the realm of transcriptomics, nanopore RNA sequencing enables comprehensive exploration of transcript isoforms and alternative splicing events, contributing to a richer understanding of gene expression and regulation. This sequencing technology has also been combined with single‐cell proteomics, providing higher sensitivity, wider throughput, digital quantification, new data modes, instrument simplification, cost reduction, and increased accessibility (Figure 6).^30^

### 3.2 Nanoomics in lipidomics

Furthermore, the research conducted by Papafi lippou and colleagues studied the interactions between NPs and lipids. In their study, they scrutinized the adsorption of lipids onto NP surfaces subsequent to plasma incubation. Their investigation led to a profound understanding of the lipid bio‐coronas that emerged on these NPs. This bio‐corona formation, facilitated by the autonomous adsorption of lipids, was found to comprise a rich diversity of molecular components.^31^ The most notable constituents of these lipid bio‐coronas were ceramides, sphingolipids, triglycerides, glycerophospholipids, fatty acids, and N‐acyl ethanolamine. These lipid species collectively impart a complex and dynamic molecular profile to the NP surfaces. Such an intricate lipid composition holds significant implications for the biological interactions and responses triggered by these NPs within the intricate milieu of the body. This exploration into the lipid bio‐coronas highlights the nuanced interplay between NPs and the biological environment. The complexity of these interactions is emphasized by the autonomous adsorption of various lipids on the surface of the NPs, demonstrating the dynamic nature of the nanobiological interface. Understanding the composition and behavior of these lipid bio‐coronas is critical for deciphering the broader effects of NP interactions within living organisms.^32^

### 3.3 Nanoomics in proteomics

**FIGURE 7:**
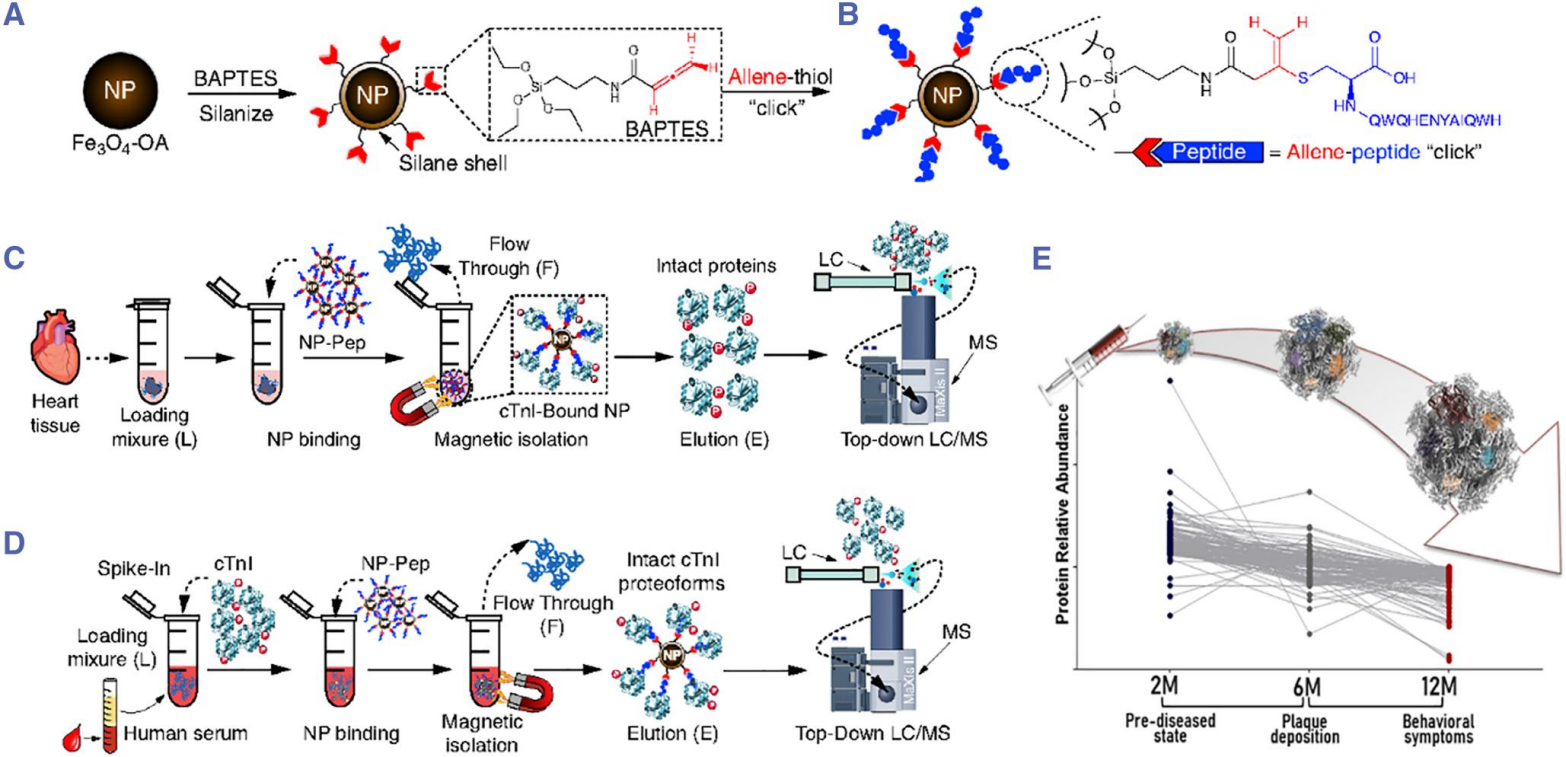
(A) Design and characterization of surface‐functionalized nanoparticles (NPs) for capturing cTnI. Silanization of Fe_3_O_4_ NPs using an allene carboxamide‐based organosilane monomer (BAPTES) for cysteine‐thiol‐specific bioconjugation. (B) Illustration of the rationally designed NPs that are surface functionalized with a 13‐mer peptide that has a high affinity for cTnI (NP‐Pep) for cTnI enrichment. The 13‐mer peptide possesses a C‐terminal cysteine that selectively reacts with the allene carboxamide moiety on the salinized NPs. (C) Evaluation of enrichment performance and reproducibility of NP‐Pep for capturing cTnI. Schematic illustration of the nanoproteomics strategy for cTnI enrichment and top‐down MS analysis of cTnI proteoforms. Heart tissue extract (loading mixture, L) was first incubated with the NP‐Pep. Following magnetic isolation, the nonspecific proteins are removed. The NPs are washed and the NP‐bound proteins of interest are eluted and analyzed by top‐down LC/MS. (D) Nanoproteomics enables comprehensive analysis of cTnI proteoforms from human serum. Nanoproteomics assay utilizing NP‐Pep for specific enrichment of cTnI from serum and subsequent top‐down MS analysis of cTnI proteoforms. cTnI was first spiked into human serum to prepare the loading mixture. The NPs were then incubated with the serum loading mixture, the cTnI‐bound NPs were magnetically isolated, and the unwanted and nonspecific proteins were removed as flow through. The captured cTnI is then eluted and the final elution fraction after enrichment is analyzed by top‐down LC/MS. *Source:* (A‐D) Reproduced under terms of the CC‐BY license.^33^ Copyright 2020, The Authors, published by Springer Nature. (E) Schematic description of the experimental design. PEGylated liposomes were intravenously injected and subsequently recovered from the blood circulation of 2, 6, and 12‐month‐old APP/PS1 and wild‐type C57 male mice. “Healthy” and “diseased” in vivo formed protein coronas were comprehensively characterized and compared by label‐free mass spectrometry (LC‐MS/MS) to identify differentially abundant proteins. *Source:* Reproduced under terms of the CC‐BY license.^34^ Copyright 2021, The Authors, published by American Chemical Society.

Nanoomics has particularly profound applications in the field of proteomics. Tiambeng et al applied nanotechnology to the detection of low‐abundance proteins in blood (Figure 7).^33^ Considering the wide dynamic range of protein concentrations in blood, often spanning over 10 orders of magnitude, detecting low‐abundance proteins requires innovative approaches. Using peptide‐functionalized superparamagnetic NPs, they directly enriched cardiac troponin I (cTnI) from serum, improving the diagnosis of cardiovascular syndrome by monitoring changes in cTnI levels. This NP‐based proteomics technique, integrated with nanotechnology, proved to be more convenient and comprehensive than traditional immunobead detection methods, as it retained intrinsic protein structural information. In the discovery of blood biomarkers for ovarian cancer through proteomics, the application of Caelyx® liposomes to incubate and purify plasma samples from healthy donors and ovarian cancer patients revealed that the liposomes maintained their original size and structure. However, proteins adhering to their surfaces formed protein coronas. Using LC‐MS/MS technology, 413 differential proteins were detected, including 72 proteins previously reported in relation to ovarian cancer. Some of these differential proteins were validated using ELISA, demonstrating the potential of this nanotechnology‐proteomics approach for identifying potential biomarker proteins.^35^ Similarly, nanotechnology provided better support for proteome profiling in Alzheimer's disease patients' blood, where disease‐specific protein coronas formed on the surface of NPs facilitated distinctive identification for proteomics analysis, while most of the varied plasma proteins exhibited an increase in abundance during the early asymptomatic phases, their levels experienced a notable decline due to amyloidosis, indicating a dynamic shift in the blood proteome specific to Alzheimer's disease based on the stage of the disease. Utilizing the nanoplatform's analytical prowess to decipher pathological shifts in the proteome unique to the disease, this non‐invasive technology holds the potential for early detection and intervention. Its implications extend not only to Alzheimer's disease patients but also to individuals afflicted by various other medical conditions.^34^, ^36^, ^37^, ^38^, ^39^

## 4 APPLICATIONS OF OMICS TECHNIQUES IN ASSESSING THE SAFETY OF NANOMATERIALS

Omics techniques as cutting‐edge technologies and tools, in the detection of the impact of nanomaterials on the human body, and extensive application has already begun. Conducted research on nanomaterials such as Silica nanoparticles (SiNPs), which have harmful effects on human health, using proteomics and metabolomics. Focusing particularly on the liver as the target organ, the research aimed to investigate the mechanisms underlying the toxicity induced by SiNPs. The results from proteomics indicated abnormal protein synthesis and misfolding as potential reasons for liver toxicity. Additionally, metabolomics results demonstrated that SiNPs alter metabolites such as glucose and alanine, leading to metabolic dysfunction.^40^ Research has demonstrated SiNPs exposure inevitably induces damage to the respiratory system. The metabolic responses of lung bronchial epithelial cells (BEAS‐2B) under SiNPs exposure indicated that oxidative stress‐ and mitochondrial dysfunction‐related GSH metabolism and pantothenate and coenzyme A biosynthesis were significantly perturbed.^41^ High‐throughput RNA sequencing (RNA‐Seq) was applied to reveal the hepatotoxicity of Nickel oxide NPs (NiO‐NPs) in liver cells. Hypoxia stress played an important role in this process.^40^ As a widely applied nanomaterial, silver nanomaterials (AgNMs) also have potential adverse biological effects. More than 30 metabolites such as glycerol, L‐leucine, and uridine were found significant regulation in the metabolic analysis. Metabolic pathways including amino acid metabolism, fatty acid metabolism, and energy metabolism like glycolysis were disturbed by AgNMs.^42^

Mesoporous silica nanoparticles (MSNs) are pharmaceutical excipients. Comparing intravenous (20 mg/kg/d) with oral administration (200 mg/kg/d) of MSNs for 10 days. The metabolic results showed that metabolites like succinate, hypoxanthine, GSSG, NADP+, NADPH, and 6‐phosphogluconic acid significantly change because of intravenous administration. At proteomic and transcriptomic levels, GPX, SOD3, G6PD, HK, and PFK increase showed that elevation of glycolysis and pentose phosphate pathway, synthesis of glutathione and nucleotides, and antioxidative pathway activity, whereas oxidative phosphorylation, TCA and mitochondrial energy metabolism were reduced. These results suggested that oral administration exhibited milder effects than intravenous injection.^43^ Nanomaterials will also have an impact on the original diseases of the human body. The presence of SiNPs had a significant impact on the hepatic metabolic profile of ApoE mice, particularly affecting amino acid and lipid metabolism.^44^ In conclusion, the impact of nanomaterials on the body remains a significant aspect that we cannot ignore.

## 5 DISCUSSION

As a pioneering interdisciplinary field, nanoomics seamlessly blends the fields of nanotechnology and omics, providing a promising catalyst for advancing omics research. The integration of these two distinct but complementary domains can revolutionize how we explore biological systems. This convergence has given rise to a novel scientific approach‐nanoomics. While the road ahead may be unknown and fraught with complexities, it is precisely this unexplored territory that inspires scientific curiosity and progress. The continuous exploration and development of nanoomics hold the promise of unveiling hidden facets of biological systems, ultimately contributing to breakthroughs in areas spanning medicine, agriculture, and environmental science.

By analyzing the changes of individual genes, proteins and metabolites, nanoomics can provide more accurate treatments and protocols for each individual.^45^, ^46^, ^47^ Currently, nanoomics mainly detects the changes of proteins to diagnose some diseases, but it has not been well applied to detect the changes of metabolites that are more phenotypic and the changes of deeper genes. With the continuous development of nanoomics, more sensitive detection of changes at the metabolic level and gene level can detect smaller changes, which has far‐reaching significance for the early diagnosis of major diseases such as cancer and whether disease recurrence occurs after recovery.^48^ The development of the disease is determined by detecting changes in substances related to the different processes of the disease, such as proteins and metabolites. Nanoomics will also have greater help in the environmental field, through the development of more sensitive sensors that can monitor the production and change of pollutants in the environment, while the relevant nanomaterials can be used to accurately detect specific trace pollutants according to specific needs. Nanoomics can also accelerate the process of drug research and development.^49^ At present, nanotechnology is mainly used to provide drug carriers for targeted drug release. However, nanoomics can further understand the distribution and metabolism of drugs in the body, accurately analyze the processes in the body and accurately grasp the target organs, so as to optimize the design of drugs. This will greatly shorten the research time of drug processes in vivo, provide a new method for drug development, and also provide a new means for discovering new organs and targets of drugs.

Although nanoomics has great potential, there are still some problems. One key issue is how to standardize the new paradigm of nanoomics that urgently needs to be solved at present. Hadjidemetriou.et al define nanoomics as the use of nanotechnology to separate analytes from biological fluids for subsequent (multiple) omics analysis,^3^ and the liquid detection of nanoomics of cancer group was discussed at the same time, the standardized process of combining nanotechnology with multi‐omics is in the perfect stage. At the same time, nanoomics is also limited by the analysis of omics data. Although the analysis of omics data is also in the stage of continuous development, there are still a large number of unknown molecules in organisms that are unknown to us, so the subsequent data analysis is also limited by the existing data analysis. Likewise, it is essential to direct our attention toward the potential detrimental impacts of nanomaterials employed in the field of nanoomics on both human health and the environment. In this context, it becomes imperative to consider the potential risks and consequences that these nanomaterials might pose.^50^, ^51^, ^52^, ^53^ Nanomaterials possess distinct physicochemical characteristics that differ from their bulk counterparts. These unique properties, while advantageous for specific applications, could potentially lead to unintended adverse effects. When these nanomaterials are in contact with biological systems, there is a possibility of interactions at the cellular and molecular levels that could influence the health of living organisms.^54^ As we venture further into the realm of nanoomics, it is vital to adopt a proactive and responsible approach. This involves thorough research to understand the potential risks associated with the use of nanomaterials and develop strategies to mitigate these risks. Regulatory frameworks should also be in place to ensure the safe and ethical application of nanomaterials in scientific research.^55^ Applications of omics techniques have had a profound impact on the assessment of nanomaterials' safety, providing valuable insights into their potential effects on biological systems. By analyzing genes, mRNAs, proteins, and metabolites affected by exposure to nanomaterials, it provides a holistic view of how cells respond to nanomaterials and their potential toxicity. Applying genomics, transcriptomics, proteomics, and metabolomics to the development of nanomaterials can provide a more comprehensive understanding of the role of nanomaterials. In summary, the unfolding chapters of nanoomics hold great promise, particularly in the realm of disease diagnosis, where new horizons are poised to emerge. This expectation is driven by the potential of nanotechnology to reshape the way we understand and manage diseases. With the convergence of nanotechnology and omics, multiple pathways are being explored that could lead to transformative breakthroughs that enrich our ability to decipher the intricacies of life and health. One of the most exciting prospects is disease diagnosis. Nanoomics promises to revolutionize the field of diagnostics by providing unprecedented insights into the molecular signature of disease. As nanotechnology allows for the manipulation of biomolecules with unprecedented precision, it promises to unearth subtle molecular clues that may indicate disease long before obvious symptoms appear. This potential for early detection is simply revolutionary because it provides the opportunity to intercept a disease in its infancy, when interventions are most effective. The integration of nanotechnology and omics gives us a powerful toolset to explore these molecular landscapes, allowing us to discover disease markers with astonishing accuracy. In addition, the combination of nanotechnology and omics has the potential to unlock the complexity of disease progression. Through this alliance, the ability to investigate the molecular events underpinning the development of the disease needs to be acquired. By carefully studying the tiny interactions between biomolecules, we can build comprehensive models of disease pathways that lead to a deeper understanding of disease mechanisms. This knowledge have revolutionary significance in tailoring treatment plans for individual patients, as designed therapies can be used to address specific molecular aberrations that drive diseases. A particularly compelling prospect is the ability of nanoomics to facilitate precision medicine. As we unravel the complexity of individual patients' molecular profiles, we gradually approach treatments that are fine‐tuned according to their unique biological makeup. This shift from a broad to an individualized approach could mean a revolution in patient care, improving treatment outcomes and minimizing adverse reactions. The precise manipulation capabilities of nanotechnology and the integration of broad molecular analysis in omics have laid the foundation for a new era of healthcare in which treatments are as unique as the patient itself.

Indeed, the future trajectory of nanoomics is full of potential. The convergence of nanotechnology and omics promises to unlock the mysteries of life and health. By enabling us to study the intricate molecular landscape of disease, nanoomics allows us to usher in an era of early detection, precise disease characterization, and personalized treatment. The profound impact of this convergence is poised to reverberate across various domains, enhancing our ability to understand, manage, and ultimately improve the well‐being of individuals. The frontier of nanoomics beckons us with the tantalizing prospect of a future where science and technology synergize to discover the secrets of life.

## AUTHOR CONTRIBUTIONS

Ruogu Qi, Jinjun Shan and Yuanyuan Guo conceived the idea; Xuan Wang and Weichen Xu wrote the manuscript and edited the figure; Jun Li and Chen Shi revised the manuscript.

## CONFLICT OF INTEREST STATEMENT

The authors declare that there are no competing interests.
